# Comparative Protective Effect of *Nigella sativa* Oil and *Vitis vinifera* Seed Oil in an Experimental Model of Isoproterenol-Induced Acute Myocardial Ischemia in Rats

**DOI:** 10.3390/molecules26113221

**Published:** 2021-05-27

**Authors:** Ioana Corina Bocsan, Raluca Maria Pop, Octavia Sabin, Elias Sarkandy, Paul-Mihai Boarescu, Ştefan Horia Roşian, Poliana Mihaela Leru, Veronica Sanda Chedea, Sonia Ancuța Socaci, Anca Dana Buzoianu

**Affiliations:** 1 Department of Pharmacology, Toxicology and Clinical Pharmacology, “Iuliu Hatieganu” University of Medicine and Pharmacy, Victor Babes, No. 8, 400012 Cluj-Napoca, Romania; bocsan.corina@umfcluj.ro (I.C.B.); octavia.sabin@umfcluj.ro (O.S.); elias.sarkandy@gmail.com (E.S.); abuzoianu@umfcluj.ro (A.D.B.); 2 Department of Pathophysiology, Iuliu Haţieganu University of Medicine and Pharmacy Cluj-Napoca, 400012 Cluj-Napoca, Romania; boarescu.paul@umfcluj.ro; 3 “Niculae Stăncioiu” Heart Institute Cluj-Napoca, 19-21 Calea Moților Street, 400001 Cluj-Napoca, Romania; dr.rosianu@gmail.com; 4 Department of Cardiology—Heart Institute, “Iuliu Haţieganu” University of Medicine and Pharmacy Cluj-Napoca, 19-21 Calea Moților Street, 400001 Cluj-Napoca, Romania; 5 Department of Family Medicine, Carol Davila University of Medicine and Pharmacy, 050474 Bucharest, Romania; polianaleru@yahoo.com; 6 Colentina Clinical Hospital, 020125 Bucharest, Romania; 7 Research Station for Viticulture and Enology Blaj (SCDVV Blaj), 515400 Blaj, Romania; chedeaveronica@yahoo.com; 8 Department of Food Science, University of Agricultural Sciences and Veterinary Medicine of Cluj-Napoca, Calea Manaștur 3-5, 400372 Cluj-Napoca, Romania; sonia.socaci@usamvcluj.ro

**Keywords:** black cumin oil, grape seed oil, cardiovascular disease, anti-inflammatory, antioxidant

## Abstract

The study’s aim was to characterize the composition of *Nigella sativa* seed (NSO) and grape seed (GSO) oils, and to evaluate their cardioprotective and anti-inflammatory effect on isoproterenol (ISO)-induced ischemia in rats. *Materials and Methods*: NSO and GSO supplements were physicochemically characterized. Liquid chromatography–mass spectrometry (HPLC-MS), Fourier-transform infrared spectroscopy (FTIR), and gas chromatography–mass spectrometry (GC-MS) analyses were used to determine the phytochemical composition in the oils. Total polyphenol content (TPC) and in vitro antioxidant activity were also determined. Pretreatment with 4 mL/kg/day NSO or GSO was administered to rats for 14 days. The experimental ischemia was induced by a single administration of ISO 45 mg/kg after 14 days. An electrocardiogram (ECG) was performed initially and 24 h after ISO. Biological evaluation was done at the end of experiment. *Results*: The HPLC-MS, GC-MS, and FTIR analyses showed that both NSO and GSO are important sources of bioactive compounds, especially catechin and phenolic acids in GSO, while NSO was enriched in flavonoids and thymol derivatives. Pretreatment with GSO and NSO significantly reduced ventricular conduction, prevented the cardiotoxic effect of ISO in ventricular myocardium, and reduced the level of proinflammatory cytokines and CK-Mb. *Conclusion*: Both NSO and GSO were shown to have an anti-inflammatory and cardioprotective effect in ISO-induced ischemia.

## 1. Introduction

Cardiovascular diseases remain one of the leading causes of morbidity and mortality worldwide, regardless of socioeconomic status [1]. Acute myocardial infarction, which is considered part of the coronary artery diseases spectrum, can be its first manifestation or can occur in its chronic evolution. Myocardial infarction is an acute condition produced by an imbalance between coronary blood supply and myocardial demand, leading to necrosis of the myocardium. Myocardial infarction is associated with an inflammatory response [2], production of oxygen-derived free radicals, and alteration of the extracellular matrix with tissue injury. All these processes lead to fibrosis and myocardial remodeling, responsible for arrhythmias and other cardiac complications [3,4]. Thus, an immediate and long-term treatment is necessary to control pathophysiological mechanisms to preserve a good function of the myocardium, to prevent the extension of myocardial lesions, and to reduce death caused by cardiovascular diseases [2].

Natural products have been used for centuries for the relief and cure of diseases. Natural products are complex and diverse, leading to numerous studies of medicinal plants and great progression in this domain in the past two decades, with more and more bioactive compounds being isolated and pharmacologically characterized. Some of these active compounds are used as drugs, either in their original or in a semisynthetic form, while others are used as natural supplements as classified under the wide umbrella of nutraceuticals [5]. Around 61% of the small-molecule drugs introduced in therapy worldwide between 1981 and 2002 were derived from natural products [6]. Thus, the discovery of new natural compounds with anti-inflammatory and antioxidant properties are still of great interest, especially for the prevention and treatment of cardiovascular diseases, representing the main cause of morbidity and mortality in the world.

*Nigella sativa* is an annual herbaceous plant that belongs to the botanical Ranunculaceae family. Its seeds contain many active compounds, with anti-inflammatory, anti-ischemic, antihypertensive, hypoglycemic, and cardioprotective effects [7,8]. Compounds from *Nigella sativa* have been shown to have effects on monocyte-derived macrophages, which are prone to take up oxidized low-density lipoprotein (LDL) and augment local inflammation. The effects of *Nigella sativa* in reducing atherosclerotic process were carried out by decreasing the level of proinflammatory mediators, released by primary macrophages. The biologically active compounds thymoquinone and carvacrol are supposedly the most important, which were demonstrated to have anti-inflammatory properties, radical-scavenging ability, and variable antioxidant activity [9,10].

*Vitis vinifera* (grapevine) is a perennial plant of the Vitaceae family widely used for grape and wine production. Grape seed oil, rich in phenolic compounds, fatty acids, and vitamins, has beneficial properties, mainly detected by in vitro studies. Its beneficial effects include the modulation of antioxidant enzyme expression, protection against oxidative damage in cells, antiatherosclerotic and anti-inflammatory effects, and protection against some cancer types [11,12]. Grape seed oil contains a large amount of phenolic compounds [11], including two stilbenes—resveratrol and piceatannol. The cardiovascular protection given by these derivates is based on the modulation of oxidative processes, inhibition of endothelial dysfunction, and induction of vascular endothelium-dependent vascular relaxation by redox regulation and nitric oxide (NO) production, thus resulting in an antiatherosclerosis effect, as well as on energy metabolism regulation, stress resistance, exercise, and fasting mimetics [13].

The aim of the present study was to characterize the main compounds identified in the oils of *Nigella sativa* seeds and *Vitis vinifera* seeds and their phenolic fraction, and to evaluate their cardioprotective effect on an animal model of ischemia induced by isoproterenol in rats.

## 2. Results

In the present study, the oils as raw materials, as well as their methanolic extracts, were characterized to have a complete overview of their composition in terms of bioactive compounds with a potential cardioprotective effect.

### 2.1. NSO and GSO Characterization

#### 2.1.1. NSO and GSO Physicochemical Properties

Some of the relevant physicochemical characteristics of NSO and GSO oil samples such as refractive index, iodine value, free acidity, and peroxide value are presented in Table 1.

These parameters, generally used as quality indicators in the case of edible oils, are in the same range as those identified in the literature for the same types of oils [14,15]. The only difference was in the case of the iodine index, having lower values in our study than those reported in the literature. This could be explained by several factors such as the technological process for obtaining the oils, the degree of maturation of the fruits and seeds from which they were extracted, or storage conditions.

#### 2.1.2. NSO and GSO Phytochemicals Characterization

**Figure 1:**
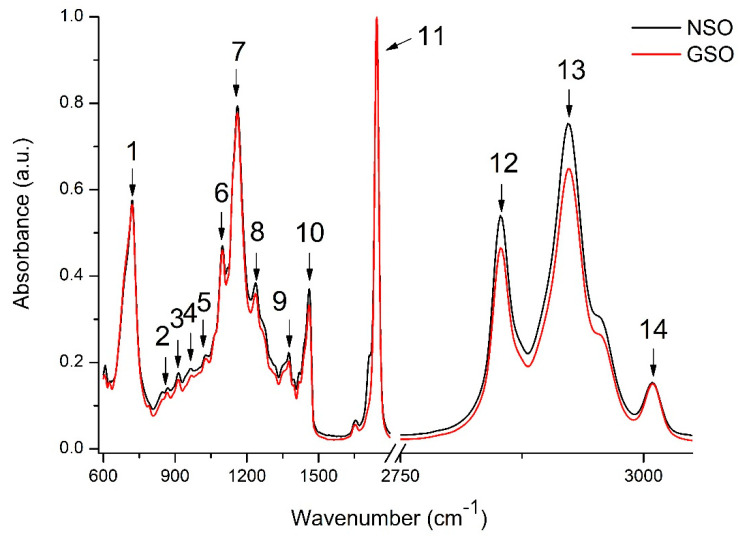
NSO and GSO general FTIR spectra (600–3100 cm^−1^). For peak assignment, see Table 2.

Figure 1 presents the general FTIR spectra (600–3100 cm^−1^) for NSO and GSO. The tentative peak assignment is shown in Table 2.

As observed in Figure 1 and Table 2, the principal absorption bands of the FTIR spectra corresponded to the functional groups characteristic for edible fats and oils (peaks 1, 7, 9, 10, 11, 12, 13, and 14) [16,17,18]. Peaks 5, 6, and 7 could also be attributed to the C–OH stretching vibrations possibly identified in flavonoids [19]. Peak 6 could also be attributed to the absorption bands in the epigallocatechin, epigallocatechin gallate, and epicatechin molecules in the oils [20]. The literature data report that the asymmetric and symmetric stretching vibration of C–O in aromatic and –OH groups in the hydrolyzable tannins can be identified in several spectral regions, e.g., 1050 to 1165 cm^−1^ and 800 to 646 cm^−1^, respectively [21] or 1326 to 1322 cm^−1^ and 1040 to 1036 cm^−1^, respectively [21,22]. For proanthocyanidins, the region between 1320 and 1230 cm^−1^ was identified [21].

Furthermore, the chemical composition of the volatile compounds from NSO and GSO was assessed by GC-MS, and the identified molecules are presented in Table 3.

The profile of volatile compounds identified in the oils is in accordance with that reported by the literature data [23,24] but with a different concentration profile. The concentration variability among samples can be explained by several factors, connected with oil extraction and the detection technique or with raw material genetic variability, origin, developmental stage, or environmental conditions. As Table 3 indicates, NSO contained α-thujene as the major compound, followed by *p*-cymene, α-pinene, and β-pinene, accounting for approximately 90% of the total compounds. Fewer compounds were identified in GSO, with hexanal and 1-butanol, 3-methyl-, acetate as the major compounds, representing approximatively 85% of total compounds.

### 2.2. NSO and GSO Extracts Characterization

The total polyphenol content of the two oils was assessed using the Folin–Ciocâlteu method. Thus, the total polyphenol content determined in the samples was 1.88 ± 0.01 mg gallic acid equivalents (GAE)/100 g NSO and 0.75 ± 0.01 mg GAE/100 g GSO. The total antioxidant activity, measured by the DPPH method, was 12,713.42 ± 156.41 mMT/100 g NSO and 1390.65 ± 1.45 mMT/100 g GSO. The results were in the range reported in the literature for both oils [25,26,27].

**Figure 2:**
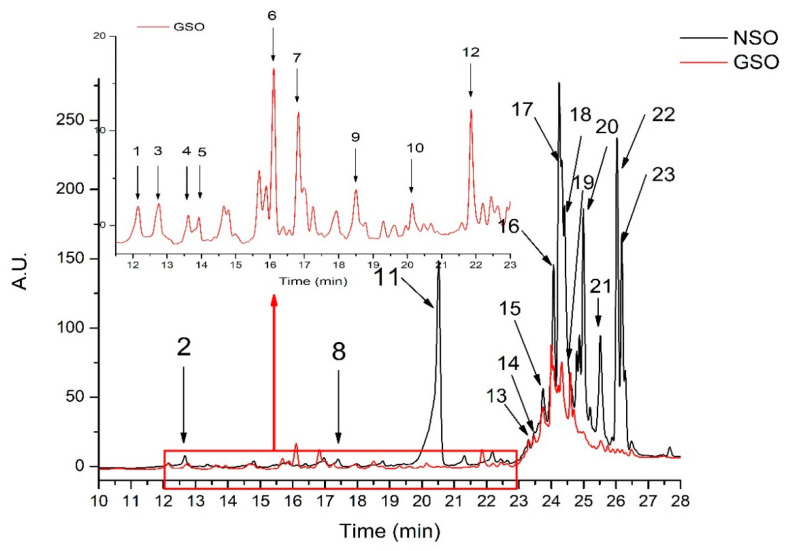
Comparative HPLC chromatograms of NSO and GSO. For peak assignment, see Table 4.

Next, the qualitative and quantitative profiles of the phenolic compounds extracted from NSO and GSO are shown in Figure 2 and Table 4. In total, 13 compounds were identified in the NSO extract and 16 were identified in the GSO extract. The identification was done by comparing compound retention times, UV/Vis absorption spectra, and the [M + H]^+^ protonated molecules with those reported in the literature [8,24,28,29,30,31,32,33]. The identified compounds in the oil extracts belonged to various classes such as hydroxybenzoic acids (peak 1, 4), pavine alkaloids (peak 2), flavan-3-ol (peak 3), hydroxycinnamic acids (peak 5, 6, 7), flavonoid glycoside (peak 8), dicaffeoylquinic acids (peak 9, 10), tannins (peak 12,14 17, 22, 23), and monoterpenoid phenol (peak 11, 20, 21) (Table 4).

The next phase of the experiment was the investigation of the cardiac effects of NSO and GSO in the prevention of ISO-induced ischemia (measured by ECG and biochemical parameters), using the 32 rats that were included in the control and experimental groups. During the experimental follow-up, all rats survived.

### 2.3. The Effect of NSO and GSO on Electrocardiogram Parameters

**Figure 3:**
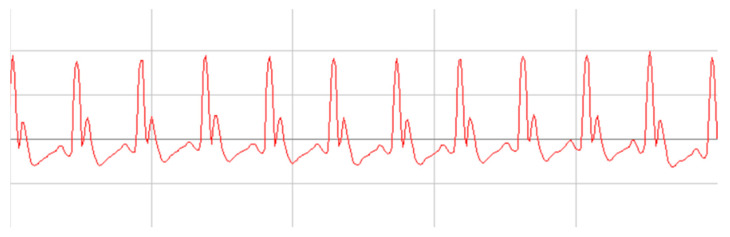
Normal EGC record from first day of experiment.

The analysis of ECG parameters recorded at baseline showed no significant differences in the experiment group (*p* > 0.005) (Table 5). A representative ECG record for all groups is shown in Figure 3.

The second ECG evaluation performed on day 14 before ISO administration did not find significant differences between animals that received NSO or GSO for 2 weeks, along with no significant variation compared to basal records (*p* > 0.005).

Heart rate was significantly increased by ISO in all experimental groups compared to baseline records (*p* < 0.001) (Table 6). ISO administration increased heart rate compared to the control group (*p* < 0.001). Pretreatment with GSO prevented the increase in heart rate induced by ISO (*p* ≤ 0.036), but this was not the case for NSO (*p* = 0.267).

**Figure 4:**
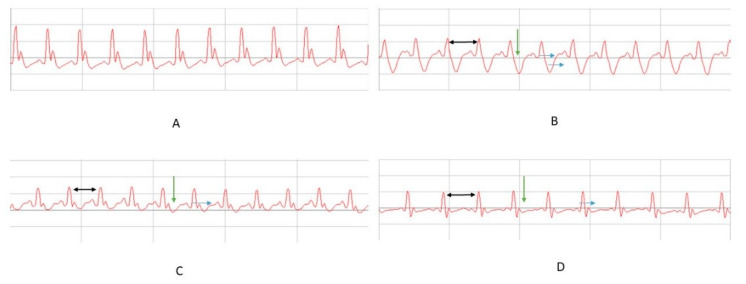
The ECG records in experimental groups with (**A**) C group, (**B**) C-ISO group, (**C**) NSO + ISO group, and (**D**) GSO + ISO on day 15 after MI induction 24 h after ISO administration (groups 2 to 4): increased RR interval (black arrow), ST-segment depression (green arrow), QT interval prolongation (blue arrow). The amplitude of these changes was different in the experimental groups.

PR, QT, and QTc intervals were increased after ISO administration compared to baseline records (*p* < 0.001), while RR interval and R wave amplitude were reduced (*p* < 0.001) (Figure 4). PR interval was increased by ISO administration compared to the control group (*p* = 0.004). Pretreatment did not prevent the increase in PR interval (NSO, *p* = 0.152; GSO, *p* = 0.115). The QRS complex was enlarged in all animals that received ISO compared to the control group (*p* < 0.001). NSO and GSO did not significantly prevent the enlargement of the complex (*p* = 0.994 and *p* = 0.204, respectively).

The increase in QT and QTc intervals was significantly reduced by NSO and GSO pretreatment compared to the positive control group treated with ISO (*p* < 0.001). There were no differences between experimental substances (*p* = 0.998, respectively *p* = 0.504).

RR interval was significantly reduced in animals that received only ISO (*p* < 0.001). If compared to group 2, after ISO administration, the animals from groups 3 and 4 showed an enlargement of the RR interval, but the enlargement was significant only in the group treated with GSO (*p* = 0.036), not in the case of NSO (*p* = 0.818). Regarding the amplitude of the R wave, it was decreased by ISO administration compared to the control group (*p* < 0.001). Both NSO and GSO significantly prevented the reduction in its amplitude (*p* < 0.001), without differences between them.

### 2.4. The Effect of NSO and GSO on Biochemical Parameters

**Figure 5:**
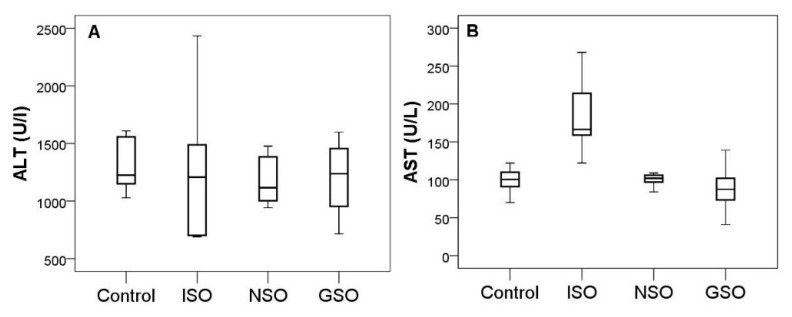
ALT (**A**) and AST (**B**) serum levels in the experimental groups.

Alanine aminotransferase (ALT) level was not influenced by the induction of myocardial infarction or by pretreatment with NSO and GSO (*p* > 0.05) (Figure 5). ALT was significantly increased by ISO administration compared to the control group (*p* = 0.001). Both NSO and GSO significantly reduced the augmentation of aspartate aminotransferase (AST) after induction of acute myocardial infarction (*p* = 0.001, respectively *p* < 0.001), without any difference between NSO and GSO (*p* = 0.785) (Figure 5).

### 2.5. The Effect of NSO and GSO on Cardiac Enzyme Activity

**Figure 6:**
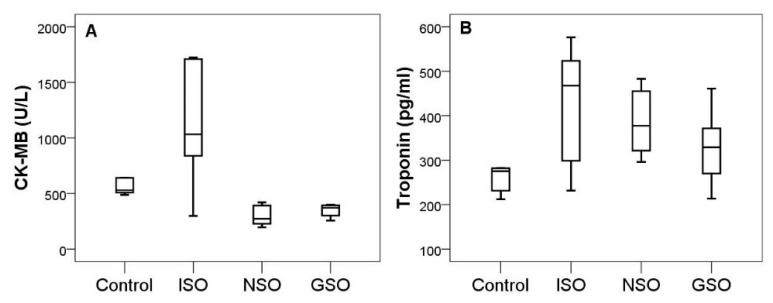
Serum levels of cardiac enzymes CK-MB (**A**) and troponin (**B**) in the experimental groups.

Troponin level was increased by ISO administration in all experimental groups (Figure 6). Pretreatment with NSO or GSO reduced augmentation of troponin, but the differences did not reach the level of statistical significance in experimental groups (*p* > 0.05). The reduction was more pronounced in group 4 that received GSO. The myocardial fraction of creatine kinase (CK-MB) significantly increased at 24 h after ISO administration (*p* = 0.001). Both NSO and GSO prevented the increase in CK-MB (*p* < 0.001), without differences between them (*p* = 0.993).

### 2.6. The Effect of NSO and GSO on Inflammatory Markers

**Figure 7:**
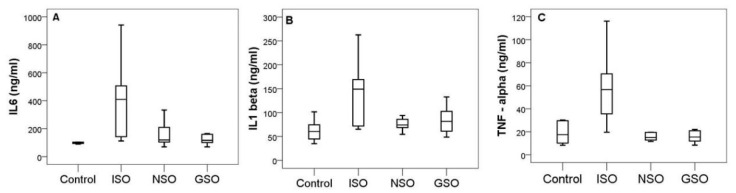
Serum levels of inflammatory markers IL6 (**A**), IL 1beta (**B**) and TNF-alpha (**C**) in the experimental groups.

Isoproterenol significantly induced an increase in proinflammatory cytokines compared to the control group (interleukin-6 (IL-6), *p* = 0.002; interleukin-1β (IL-1 β), *p* = 0.03, and tumor necrosis factor-alpha (TNF-α), *p* < 0.001) (Figure 7). NSO significantly decreased the upregulation of IL-1β (*p* = 0.024), IL-6 (*p* = 0.016), and TNF-α (*p* < 0.001) compared to the positive control group that received only ISO. Similar results were also observed in animals treated with GSO, which significantly reduced the inflammatory markers IL-6 (*p* = 0.005), IL-1β (*p* = 0.0047), and TNF-α (*p* < 0.001). There were no significant differences between NSO and GSO in reducing proinflammatory markers (*p* > 0.005 in all comparisons).

## 3. Discussion

Isoproterenol, a nonselective beta-agonist, administered in high doses, causes severe biochemical, functional, and histological alterations in the heart, comparable with those taking place in human myocardial infarction [3,34]. These changes are consequences of oxidative stress and inflammation, which alter tissue antioxidative defense systems in the myocardium [34,35,36]. Considering the above, antioxidant and anti-inflammatory substances may protect myocardial cells from myocardial infarction damage [34,37].

The isoproterenol ischemic model is characterized by reduced RR interval and increased heart rate [3,38]. It also prolongs the QRS complex, increases QT and QTc intervals, increases duration of the PR segment, and reduces R wave amplitude, thus representing ECG changes that reflect the occurrence of myocardial infarction and conduction disturbances of impulses secondarily to ischemia and due to ISO’s cardiotoxic effect [39,40]. Furthermore, the reduction in R wave amplitude reflects a low conduction due to an inflammatory edema associated with ischemia [3]. A prolongation of PR interval reflects a slow conduction in the AV node or a fibrosis in this structure [39]. Isoproterenol also significantly increases cardiac enzymes and proinflammatory markers, sustaining the role of inflammation in cardiac ischemic lesions. Isoproterenol-induced ECG changes specific to acute myocardial infarction could be a result of the loss of action potential in the myocardial cell membrane, because of oxidative stress and inflammation [3].

Pretreatment with GSO and NSO did not influence the duration of the QRS complex or PR interval, but reduced the prolongation of QT and QTc intervals and prevented the reduction in R wave amplitude induced by ISO. This is an argument for the assumption that both investigated oils do not affect the atrioventricular conduction, but partially influence ventricular conduction, thereby preventing the cardiotoxic effect of ISO. GSO, but not NSO, prevented the heart rate increase and RR interval reduction induced by ISO, which may contribute to the cardioprotection conferred by grapes products in acute ischemia.

Few published data investigated the role of NSO and GSO in myocardial ischemia. Most studies evaluated the atherosclerotic process or separately evaluated the pharmacological effects of their main constituents, i.e., thymoquinone from *Nigella sativa* [41] and resveratrol from grapes [42]. For example, long-term treatment with NSO or methanolic extracts (8–12 weeks) reduced atherosclerotic plaque formation in coronary arteries [43,44] and, through this mechanism, showed a possible protective effect in coronary artery disease, by preventing acute myocardial ischemia. In producing these effects, different mechanisms are supposedly implicated, involving serotonergic, muscarinic, and adrenergic systems [41]. In the present experiment, the heart rate was increased, while RR the interval was reduced by ISO, whereas NSO could not prevent these effects. In Xiao’s study [45], thymoquinone, NSO’s main constituent, significantly reduced heart rate in a similar experimental model, which might validate the hypothesis of a complex mechanism reported by Ojha et al. [41]. Pretreatment with NSO did not influence QRS complex duration and PR intervals, highlighting the hypothesis that NSO could have a preventative atherosclerosis effect rather than preventing the acute consequences of a beta stimulation of the heart.

Compared to NSO, GSO significantly prevented the heart rate increase and RR interval reduction induced by ISO, which may contribute to the cardioprotection conferred by grapes in acute ischemia.

GSO, like NSO, did not influence the QRS complex and PR interval. In a previous study, Badavi et al. showed that grape seed extract did not influence the heart rate and blood pressure [46], while Tiwari et al. showed that only myricetin, a flavonoid compound extracted from red wine, administered for 21 days significantly inhibited the effects of ISO on heart rate and ECG changes, as reported in the present study [47].

Both NSO and GSO reduced the prolongation of the QT interval and its corresponding value of QTc. Another possible mechanism underlying the cardioprotective effect of *Nigella sativa* could be calcium blockade [48]. Calcium is essential in maintaining the plateau phase of the action potential, and it is a potential contributor to QT interval duration. In the present study, NSO significantly reduced the prolongation of the QT interval in acute myocardial infarction, which might be related to a potential calcium blockade. The cardioprotective effect of GSO was also linked with calcium loading in the myocardium in another two models of cardiotoxicity, induced by doxorubicin [49] and by a high-fat diet [50]. We may raise the hypothesis that the NSO and GSO cardioprotective effects on acute ischemia could be partially related to calcium concentration in the myocardium. Further studies are needed to better understand the exact mechanism of action of NSO and GSO, as well as of their main constituents.

In Al Assom et al.’s study, long-term treatment with *Nigella sativa* induced coronary angiogenesis and had no effect on inducible NO synthase level, demonstrating a possible cardioprotective effect [51]. Olas et al. reported that GSO decreased platelet adhesion in vitro, and the effect was more intense than after resveratrol [52]. Sano et al. showed that GSO reduced oxidized LDL in 61 healthy subjects, suggesting its cardioprotective potential [53]. According to these observations, we suppose that both NSO and GSO may have a cardioprotective effect by modulating endothelial function and platelet aggregation rather than by reducing adrenergic stimulation of the heart.

On the other hand, thymol induced a dose-dependent negative inotropic action on the isolated heart. El-Tahir et al. demonstrated that *Nigella sativa*’s chronotropic and hypotensive effects were mediated centrally either directly or indirectly via mechanisms involving serotoninergic and muscarinic receptors [54].

Increasing evidence supports that cardiovascular diseases have an inflammatory component in their pathophysiology [55]. Several studies have described an increased expression of proinflammatory cytokines IL-1β, IL-6, and TNF-α in ISO-induced myocardial infarction [3,35,55,56]. Beta-blockers have a beneficial effect on myocardial injury, reducing the myocardial expression of proinflammatory cytokines IL-1β, IL-6, and TNF-α [41,57]; thus, proinflammatory cytokines can be used as markers to evaluate the cardioprotective effect of some natural products. Proinflammatory cytokines act as pleiotropic polypeptides that are independently associated with inflammation and oxidative stress and the release of these cytokines leads to myocardial injury through several mechanisms [55].

In the present study, a significant increase in plasmatic levels of IL-1 β, IL-6, and TNF-α was noted after administration of ISO, in agreement with previous studies [3,35,55,56]. Pretreatment with GSO and NSO significantly reduced the level of proinflammatory cytokines, suggesting a clear anti-inflammatory effect in the ischemic heart. The reduction was more intense in the group with NSO pretreatment than that with GSO pretreatment, for IL-1β and TNF-α.

Similar results were also reported by Ojha et al. for thymoquinone pretreatment in acute ischemia induced by ISO. We may assume that the anti-inflammatory effect of NSO in ISO-induced acute ischemia is a consequence of its main component thymoquinone. Twenty-eight days of pretreatment with grape seed methanolic extract reduced the myocardial expression and levels of IL-1β, Il-6, and TNF-α and decreased the oxidative stress in the infarcted and non-infarcted heart of diabetic rats, suggesting that the cardioprotective effects of grapes are a consequence of anti-inflammatory and antioxidant properties [58]. In the present study, the authors noticed the same reduction in proinflammatory cytokines, but in the serum not in the myocardium, after 14 weeks of therapy with GSO, supporting the anti-inflammatory effect of grapes. A 2 week supplementation with red wine grape pomace reduced premature death, changed TNF-α and IL-10 levels, increased plasma antioxidant activity, and attenuated myocardial infarction and dysfunction, confirming the cardioprotective effect of red grape products in ischemic heart disease [59]. We may conclude that both GSO and NSO exerted their cardioprotective effects partially through their anti-inflammatory actions.

Cardiac tropinins and serum CK-MB are used for the diagnosis of myocardial injury [60]. In the present study, the serum levels of T-troponin and CK-MB were significantly increased in 24 h after the onset of ISO-induced myocardial infarction. Pretreatment with GSO and NSO could not prevent myocardial damage and the corresponding increase in T-troponin, but it significantly reduced CK-MB. These results are different from previously published data. Danaei et al. reported a reduction in troponin level in dianozin-induced cardiotoxicity by thymoquinone treatment for 28 days [61]. The different results could be explained by the use of ISO in inducing the ischemia, which is another type of experimental model, as well as by the use of oil in the present study as compared with its main constituent thymoquinone in Danaei’s study. Higher doses are probably needed to obtain an optimal concentration of thymoquinone to prevent myocardial cell damage. Regarding the effect of GSO on troponin level, our results also do not conform to previously published data. A significant reduction in troponin-I and CK-MB levels was reported by Giribabu et al., who used diabetic rats that received ISO after 28 days of pretreatment with methanolic grape extract [58]. By comparison, we determined the level of T-troponin involved in myocardial contraction, not I-troponin, and the duration of therapy was lower. Sun et al. reported that only resveratrol 100 mg/kg and resveratrol nanoparticles conferred a cardioprotective effect by reducing cardiac troponin T (cTnT) levels after ISO-induced ischemia [62].

Previously published data sustained the hypothesis that both NSO and GSO may have cardioprotective effects, but the mechanisms are more complex and depend also on the type of extract, the isolated active compounds, and the route of administration, which might influence their bioavailability. On the basis of these results, we may assume that higher doses of these dietary supplements, over a longer term, are needed to have more consistent results in terms of cardiac damage prevention. We did not evaluate their pharmacokinetics to verify whether obtaining higher levels of active compounds would lead to a different result. In the present study, the complex analysis of NSO revealed the presence of high concentrations of flavonoids and thymol derivatives, while thymoquinone represented only 1.9% of the bioactive compounds identified in NSO. The analysis of GSO identified more catechin and phenolic acids than in the case of NSO. The most notable bioactive property of phenolic compounds is their antioxidative and anti-inflammatory capacities, as demonstrated in the present study. Xia et al. reported that grape seed possesses the greatest antioxidant activity, which is related to its high content of gallic acid, catechin, epicatechin, procyanidins, and proanthocyanidins and their synergistic effect with phenolic compounds [63]. This hypothesis could be sustained by the present study. Even though the total phenolic content was lower for GSO than for NSO, the cardioprotective effect of GSO was more pronounced, and we may assume that this effect is a consequence of the synergistic effects of catechin, procyanidins, and phenols identified in the used GSO. In contrast, the anti-inflammatory effect was more intense in the case of NSO, and this is probably related to the thymoquinone and thymol derivatives in combination with phenols. Previously published data revealed that both thymoquinone and thymol possess anti-inflammatory activities. We may conclude that in vitro activities do not always correlate directly with in vivo effects, and natural supplements should be analyzed as a function of their total complexity, because interactions between active biocompounds may lead to more significant clinical effects.

The main strength of this study was the comparative analysis of the cardioprotective and anti-inflammatory effects of two natural supplements, as entire compounds, in ISO-induced experimental ischemia. This study also had some limitations. Firstly, a histological examination to confirm the size of ischemic lesions on the myocardium was not performed. Secondly, only the in vitro antioxidant effect of NSO and GSO was determined.

## 4. Materials and Methods

### 4.1. Chemicals

Acetonitrile, methanol, and acetic acid were purchased from Merck (Darmstadt, Germany). All other chemicals used for physicochemical oils characterization and sample extractions were of analytical grade and were purchased from Sigma-Aldrich (Steinheim, Germany). Water and isoprenaline hydrochloride (98%) for acute myocardial infarction induction were purchased from Sigma–Aldrich (St. Louis, MO, USA). Commercial dietary supplements, AquaNano Negriol in cold-pressed oil form, extracted from *Nigella Sativa* seeds, and Solaris grape oil, from the cold-pressed seed of *Vitis vinifera*, were purchased from the pharmacy and used as experimental substances.

### 4.2. Oil Physicochemical Characterization

For the physicochemical characterization, the following parameters were investigated: the refractive index, the iodine index, the free acidity, and the peroxide value.

#### 4.2.1. Refractive Index

The refractive index was directly determined with the Abbe Refractometer using the apparatus according to the Association of Official Analytical Chemists methods. The refractive index is directly proportional to the degree of unsaturation and is also affected by the oxidation, free fatty-acid content, and thermal treatment [64,65].

#### 4.2.2. Iodine Index

The iodine index, free acidity, and peroxide value were determined by titrimetry. The iodine index gives information on the unsaturation degree. The double bonds in fat molecules react with iodine. The amount of iodine in grams consumed by 100 g of oil represents a measure of oil and fat unsaturation. The determination was based on American Oil Chemists’ Society methods [65].

#### 4.2.3. Free Acidity

Free acidity represents the number of milligrams of potassium hydroxide needed to neutralize the free acids found in 1 g of sample. The determination was based on American Oil Chemists’ Society methods [65].

#### 4.2.4. Peroxide Values

Peroxide values give information regarding an oil’s incipient rancidity and conservation state. It is expressed by the number of milliliters of sodium thiosulfate (0.002 N) consumed by 1 g oil according to American Oil Chemists’ Society methods [65].

### 4.3. Oil Phytochemical Characterization

The FTIR analysis and in-tube extraction technique (ITEX) coupled with GC-MS analysis was used to determine the phytochemical composition of the oils as described in Pop et al. [8].

#### 4.3.1. FTIR Analysis

The FTIR spectra of NSO and GSO were measured using a Shimatzu IR Prestige-21 (FTIR) spectrometer. The oils were measured directly on the attenuated total reflectance crystal between 4000 and 650 cm^−1^. Between measurements, the crystal was cleaned with acetone. The identification of specific IR frequencies of functional groups was done in accordance with literature data.

#### 4.3.2. ITEX–GC-MS Analysis

ITEX–GC-MS was performed using a GC-MS Shimadzu model QP-2010 (Shimadzu Scientific Instruments, Kyoto, Japan) on a ZB-5 ms, 30 m × 0.25 mm i.d. × 0.25 µm capillary column (Phenomenex, Torrance, CA, USA). The volatile oil extraction was done using 0.2 g of oil placed in a headspace vial at 60 °C under continuous stirring (500 rpm) for 20 min. The results were expressed as a percentage of total peak area. The tentative compound identification was performed by comparing the mass spectra and fragmentation patterns of the compounds with those indicated by the software’s NIST27 and NIST147 mass spectral libraries. The retention indices were also compared with those indicated by the websites www.pherobase.com (accessed on 12 January 2021) or www.flavornet.org (accessed on 12 January 2021), taking into consideration columns with a similar stationary phase. A minimum of 85% similarity was taken into consideration.

### 4.4. Oil Extraction

The phenolic compounds in NSO and GSO (2.5 g each) were extracted using 3 mL of *n*-hexane and 4 mL of methanol/water solution (60:40; *v*/*v*). The mixture was vortexed and further sonicated for 15 min using a water bath. Afterward, the samples were centrifuged at 8000 rpm for 5 min. The methanolic phase was further collected and washed three times with 4 mL hexane using the same procedure as described above (vortex and centrifugation). Lastly, the mixture was evaporated to dryness by nitrogen flushing. Right before the quantitative and qualitative determinations, the residue was resuspended in 1 mL of MeOH.

### 4.5. Oil Extract Characterization

The oil extracts were characterized quantitatively regarding their TPC and antioxidant activity and qualitatively to identify the characteristic phenolic compound composition as previously described [8].

#### 4.5.1. Total Polyphenol Content

TPC was determined using the Folin–Ciocâlteu method [66]. The results were expressed as GAE in mg/100 g oil. Triplicate analysis was performed for each oil extract. The results of triplicate analysis were expressed using their mean values ± standard deviations.

#### 4.5.2. Antioxidant Activity

The antioxidant activity test as determined by the DPPH radical-scavenging activity of the oil extracts was evaluated following the Brand-Williams method [67]. The results were expressed as mM Trolox/100 g oil. The results of triplicate analysis were expressed using their mean values ± standard deviations.

#### 4.5.3. HPLC-MS Analysis

The liquid chromatography–diode array detection–electrospray ionization mass spectrometry (HPLC–DAD–ESI MS) method was used to identify the phenolic compounds as described in Pop et al., 2020. An Eclipse XDB C18 column (4.6 × 150 mm, 5 µm particle size) (Agilent Technologies, Santa Clara, CA, USA) was used for separation. A gradient elution of two mobile phases was used for compound separation. Mobile phase A was 0.1% acetic acid/acetonitrile (99:1) in distilled water (*v*/*v*), while mobile phase B consisted of 0.1% acetic acid in acetonitrile (*v*/*v*). The mass spectra of the investigated molecules were scanned from 100 to 1000 *m*/*z*. The NSO and GSO compounds were expressed as GAEs (*R^2^* = 0.99). Triplicate analysis was performed. The compound identification was performed by comparing specific UV/visible spectra, retention time, and mass spectra with authentic standards (when available) and literature data.

### 4.6. Animals

The protocol of the experiment was approved by the Ethics Committee of the “Iuliu Hațieganu” University of Medicine and Pharmacy Cluj-Napoca (3850/2020) and by the Sanitary-Veterinary and Food Safety Directorate from Cluj-Napoca (206/01.04.2020), following the Helsinki Declaration on Animal Studies. The national and international guidelines referring to animal care and use were followed.

Thirty-two Wistar-Bratislava male rats were randomly divided into four groups of eight animals. The animals with a body weight between 200 and 260 g were kept for the entire experiment at the Biobase of “Iuliu Haţieganu” University of Medicine and Pharmacy Cluj-Napoca. During the experiment, the animals were kept in standard conditions for accommodation, in polypropylene cages, which were acclimated at standard environmental conditions of 22–24 °C, humidity 55% ± 15%, and a 12 h/12 h light/dark cycle. All animals had ad libitum access to water and standard pellets (Cantacuzino Institute, Bucharest, Romania). Two hours before the experiment, the rats were not fed, but water was available ad libitum.

### 4.7. Experimental Protocol of acute Myocardial Infarction

The rats were randomly divided into four groups of eight animals. The animals received saline solution, NSO, or GSO for 14 days as follows:

- Group 1 (Control group)—saline solution 0.4 mL/100 g;
- Group 2 (ISO)—saline solution 0.4 mL/100 g;
- Group 3 (NSO)—*Nigella sativa* seed oil 0.4 mL/100 g;
- Group 4 (GSO)—Grape seed oil 0.4 mL/100 g.

Saline solution and experimental substances were administered orally by gavage.

The animals from groups 2–4 subcutaneously received a single dose of ISO (45 mg/kg) on day 14 of the experiment. The dose that was used was previously tested and considered the minimal dose producing acute myocardial infarction with elevation of cardiac enzymes, such as troponin [3,68].

At the end of the experiment, the rats were sacrificed by an overdose of anesthetics.

### 4.8. Electrocardiography

ECG was recorded initially at the beginning of the experiment, on day 14 before ISO administration and 24 h after ISO-induced myocardial infarction, according to a previously described protocol. Intraperitoneal injections of ketamine (26 mg/kg) and xylazine (2.6 mg/kg) were used to anesthetize the rats. To bind the electrodes on the paw pads of the rat, the animals were fixed in a supine position on a board, 15 min after the induction of anesthesia. From there, at lead II (right forelimb to left hind limb), the ECG was recorded using a Biopac MP36 system.

Calibration at 1 mV/1 cm and a speed of 50 mm/s were set for the ECG apparatus [39]. The Biopac Student Lab 3.7.7 software was used to calculate the RR and QT intervals (ms), PR segment (ms), QRS complex duration (ms), and R wave amplitude (mV) [3] from the ECG recordings.

Furthermore, the heart rate of each rat was calculated from the given RR intervals. For that, the following formula was used: HR = 60,000/RR. In addition, the Bazett formula aided with the calculation of the corrected QT intervals (QTc in ms) [69].

### 4.9. Biologic Evaluation

The blood samples were taken from retroorbital plexus at the end of the experiment. One milliliter of blood on anticoagulants was preserved, and the serum was obtained through centrifugation within first hour. The obtained serum samples were kept at −80 °C until determinations were performed.

The serum levels of AST, ALT, and CK-Mb were determined through a spectrophotometric method using an automatic analyzer Applied Biosystem (Costa Brava, Barcelona (Spain)).

The serum levels of the inflammatory cytokines TNF-α, IL-6, and IL-1β were measured using the ELISA Stat Fax 303 Plus Microstrip Reader (Minneapolis, MN, USA) with commercially available kits (rat TNF-α, IL-6, and IL-1β ABTS ELISA Development kits, PeproTech EC, Ltd., London, UK). Troponin was also measured using an Elabscience ELISA kit. The determinations were done according to the manufacturer’s instructions. For each assay, samples were diluted as needed, and protein levels were calculated using a four-parameter logistic (4-PL) curve fit.

### 4.10. Statistical Analysis

The statistical analysis was performed using SPSS version 19 (Chicago, IL, USA). Data were labeled as continuous variables. Normal distribution for continuous variables was determined using the Kolmogorov–Smirnov test. The results were expressed as the mean and standard deviation (for variables with a normal distribution) or as the median and 25th–75th percentiles (for variables with non-normal distribution). We used one-way ANOVA with Tukey correction and Spearman’s rho correlation coefficient for univariate analysis of continuous variables. The level of statistical significance was set at *p* < 0.05.

## 5. Conclusions

In the present study, the investigated dietary supplements, NSO and GSO, partially prevented ECG alterations and the modification of biological and inflammatory parameters after ISO-induced myocardial infarction. The effects on ECG changes were more pronounced in animals treated with GSO. Both NSO and GSO were shown to have an anti-inflammatory and cardioprotective effect in ISO-induced ischemia. Both compounds were shown to have good potential for future treatment options in cardiovascular diseases.

## Figures and Tables

**Table 1:** Physicochemical properties of NSO and GSO.

No	Sample	Refractive Index	Iodine Index g I_2_/100 g Oil	Free Acidity (%)	Peroxide Value O_2_, mmol·kg^−1^
1	NSO	1.466	70	1.2	<10
2	GSO	1.478	67	4.4	<10

**Table 2:** The tentative peak assignment for the general FTIR spectra (600–3100 cm^−1^) for NSO and GSO.

No	Peak (cm^−1^)	Peak Intensity		Tentative Assignment
		NSO	GSO	
1	719	0.575	0.565	CH=CH– bending out of plane
2	866	0.141	0.131	=CH_2_ wagging
3	914	0.176	0.161	–C–H bending out of plane
4	968	0.186	0.170	*trans* –CH=CH– bending out of plane
5	1028	0.217	0.209	–C–O stretch
6	1097	0.470	0.460	–C–O stretch
7	1161	0.794	0.776	–C–O stretch; –CH_2_ bending
8	1236	0.384	0.360	–C–O stretch
9	1375	0.223	0.203	–C–H bending
10	1460	0.369	0.332	–CH_2_ bending
11	1743	1	1	C=O stretching
12	2852	0.54	0.465	–CH_2_ asymmetrical stretching
13	2924	0.753	0.648	–CH_2_ symmetrical stretching
14	3008	0.154	0.151	(*trans* =C–H stretch)

**Table 3:** Gas chromatography coupled with mass spectroscopy (GS–MS) chemical composition of volatile compounds identified in NSO and GSO.

No	Compounds	Retention Time	Concentration % of Total Peak Area	
			NSO	GSO
1	Hexanal	4.024	0.98	36.68
2	1-Butanol, 3-methyl-, acetate	5.995	-	48.55
3	α-Thujene	7.61	42.97	-
4	α-Pinene	7.853	8.25	3.16
5	Camphene	8.435	0.06	-
6	Sabinene	9.258	2.38	-
7	β-Pinene	9.439	4.96	-
9	Furan, 2-pentyl-	9.907	-	0.43
10	Hexanoic acid, ethyl ester	10.234	-	8.2
12	α-Terpinene	10.912	0.27	-
13	*p*-Cymene	11.227	33.71	-
14	d-Limonene	11.383	2.07	0.75
15	Eucalyptol	11.502	0.06	-
16	γ-Terpinene	12.504	0.64	-
17	Terpinolene	13.56	0.06	-
21	Octanoic acid, ethyl ester	17.874	-	1.34
22	Thymoquinone	19.84	1.9	-
23	Cuminone	20.651	0.4	-

**Table 4:** Tentative identification, characterization, and concentration of major compounds identified in NSO and GSO extracts.

Peak No.	R_t_ (min)	UV λ_max_ (nm)	[M + H]^+^ (*m*/*z*)	Compound	Concentration μg/mL Oil	
					NSO	GSO
1	12.15	275	139	*p*-Hydroxybenzoic acid	0.807	0.971
2	12.66	260	342	Norargemonine	1.167	-
3	12.75	280	291	Catechin	-	0.987
4	13.61	280	169	Vanillic acid	-	0.799
5	13.92	320	181	Caffeic acid	-	0.807
6	16.10	321	165	*p*-Coumaric acid	-	1.749
7	16.82	322	195	Ferulic acid	-	1.519
8	17.40	350, 260	755	Kaempferol-rhamnoside-diglucoside	-	1.118
9	18.77	320	517	Dicaffeoylquinic acid	-	1.110
10	20.13	320	517	Dicaffeoylquinic acid	-	0.872
11	20.51	290	194	Thymol derivative	17.105	-
12	21.86	280	867, 291	Procyanidin trimer possibly C2 (Catechin derivative)	-	1.413
13	23.29	350, 260	755	K	2.191	-
14	23.4	280	867, 291	Procyanidin trimer (Catechin derivative)	-	2.281
15	23.75	280	375	Hydroxymatairesinol	6.687	5.852
16	24.00	280	358	Matairesinol	10.692	4.337
17	24.21	280	1099, 1085	Tanin	14.656	2.748
18	24.32	280	375	Isohydroxymatairesinol	12.076	6.990
19	24.59	280	1120	Tanin (Catechin derivative)	3.927	3.878
20	24.99	290	150	Tymol	10.561	-
21	25.51	280	414	Tymol derivative	8.382	-
22	26.03	280	1142	Tanin	11.364	-
23	26.18	280	1040	Tanin	7.645	-

**Table 5:** Basal values of ECG parameters recorded at the beginning of the experiment. C (control group), C-ISO (ISO group), NSO + ISO (*Nigella sativa* seed oil and ISO group), GSO + ISO (grape seed oil and ISO group), HR (heart rate).

Group	HR (Beats/min)	RR (ms)	PR	QRS	QT	QTc	R
C	282 ± 19	223 ± 17	42 ± 2	34 ± 2	78 ± 3	65 ± 3	2.1 ± 0.1
C-ISO	287 ± 19	237 ± 16	41 ± 2	34 ± 4	78 ± 4	65 ± 3	2.1 ± 0.1
NSO + ISO	288 ± 15	225 ± 11	42 ± 2	35 ± 4	80 ± 4	65 ± 4	2.1 ± 0.1
GSO + ISO	283 ± 16	230 ± 9	42 ± 2	35 ± 4	78 ± 4	64 ± 3	2.1 ± 0.1

**Table 6:** ECG parameters recorded after MI (day 14). C (control group), C-ISO (ISO group), NSO + ISO (*Nigella sativa* seed oil and ISO group), GSO + ISO (grape seed oil and ISO group), HR (heart rate).

Group	HR (Beats/min)	RR (ms)	PR	QRS	QT	QTc	R
C	271 ± 18	220 ± 16	42 ± 2	34 ± 2	78 ± 3	634 ± 4	2.1 ± 0.1
C-ISO	329 ± 15	186 ± 9	45 ± 2	53 ± 4	104 ± 63	94 ± 6	0.8 ± 0.1
NSO + ISO	315 ± 6	190 ± 4	43 ± 2	53 ± 4	95 ± 4	85 ± 3	1.1 ± 0.1
GSO + ISO	299 ± 15	201 ± 11	43 ± 2	49 ± 7	95 ± 4	82 ± 4	1.2 ± 0.1

## Sample Availability

Samples of the compounds NSO and GSO are available from the authors.
